# Does institutional quality complement the relationship between ownership structure and corporate social responsibility?

**DOI:** 10.1016/j.heliyon.2024.e31994

**Published:** 2024-05-28

**Authors:** Shuaib Ali, Rongwu Zhang, Muhammad Talha, Zahid Ali

**Affiliations:** a School of Management, Guangzhou University, Guangzhou, PR China; b Zhongnan University of Economics and Law, Wuhan, PR China; c Department of Commerce and Management Sciences, University of Malakand, Pakistan

**Keywords:** Institutional quality, Ownership structure, Corporate social responsibility, CEO duality, PCA

## Abstract

The key purpose of the Study is to examine if institutional quality complements the relationship between Ownership Structure and Corporate Social Responsibility disclosure and performance in the light of legitimacy and agency theory. To the best of my knowledge, it is the first study in literature of finance. The sample comprises of 112 top-performing listed firms (based on market capitalization) at Pakistan Stock Exchange from 2010 to 2019. Institutional quality comprised of world governance indicators which is developed via principal component analysis, an instrumental variable approach and content analysis are used for CSR Disclosure Index to demonstrate the relationship between ownership structure and CSR. The resources complementary phenomenon is adopted to examine the institutional quality's role. Our results show significantly positive impact of Institutional and Foreign Ownerships on CSR while negative significant influence of CEO Duality and Family Ownership on CSR, suggesting that well governed firms will be more socially responsible. In addition, the findings suggest the institutional quality's positive moderating role on the relationship between ownership structure and CSR, signifying the institutional quality's complementary role for the weak corporate environment in Pakistan. Our findings are robust to a series of tests by using Generalized Method of Moment (GMM).

## 1 Introduction

Corporate Social Responsibility (CSR) is described as the moral obligation by corporations and contribution to financial growth while the prosperous life's quality of the staff and their relatives as well as of the society [1]. Policymakers and academics have given considerable attention to CSR and its reporting in recent years. several theoretical reinforcements increasingly suggests that corporate social responsibility is a significant driver that benefits both trade and society [2,3]. Corporate social responsibility (CSR) considers the impact of a company's performance on the environment and people while maintaining profitability [4,5]. Similarly, CSR activities may help a company's brand, reputation, and capacity to draw in new clients, Thus, businesses' profitability may rise [6,7].

CEO Duality is when the Chief Executive and Chairman are the same person [8,9]. “According to Mbate [10] The term "CEO duality" describes a scenario in which the CEO simultaneously holds the position of chairman of the board”. Some prior studies argued duality negatively affects firm permeance and disclosure of information in Pakistan [11]. In Pakistan, corporates have faced incomparable challenges, providing them numerous opportunities to grow since the beginning family ownership has been quite a phenomenon in Pakistan as significant industrial power was in the hands of 22 super rich and powerful families and undoubtedly, family-controlled businesses have flourished a lot [12]. So, studying CSR in the context of ownership structure could be interesting in the perception of emerging market such as Pakistan.

Formal and casual behavior of public in a state is the IQ of that territory [13]. The regular mechanisms are the policies and principles, the structure for investors’ protection and property rights, and the managerial organization of the state. In distinction, the usual behavior of the people and civilization is informal component, which has been developed according to the antique design of behavior. Krishnan and Teo [14] stated that institutes are social miracle because they set guidelines for the game, which is compulsory for companies and administrations to tolerate.

The study complements the prior literature by considering a sample (based on market capitalization) of the non-financial Pakistani firms. According to my knowledge, it is the first study to examine the moderating impact of Institutional Quality index on the association between ownership structure and CSR and to develop Institutional Quality index via (PCA). This study donates to the prior literature of CSR, corporate governance and institutional Quality, in numerous ways; particularly, few studies from developing countries have also replicated such studies in the financial sectors of their countries [15,16]. A reason for the selection of the financial sector by previous studies could be that banks and other investment institutions pay more attention to CSR disclosure and reporting, which makes it easier for the researchers to extract the data. Moreover, this study is not limited to CSR disclosure as many past researchers have focused on that area. This study has used CSR donation as a proxy in addition to the disclosure because, in Pakistan, firms usually perceive donation as CSR which designs the study according to the context of Pakistan.

**Fig. 1:**
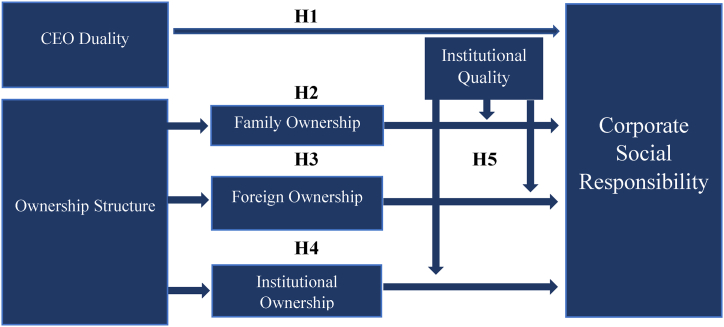
Conceptual framework.

The sample comprises of 112 top-performing listed firms (based on market capitalization) at Pakistan Stock Exchange from 2010 to 2019. Institutional quality comprised of world governance indicators which is developed via principal component analysis, an instrumental variable approach and content analysis are used for CSR Disclosure Index to demonstrate the relationship between ownership structure and CSR. The resources complementary phenomenon is adopted to examine the institutional quality's role. Our results show significantly positive impact of Institutional and Foreign Ownerships on CSR while negative significant influence of CEO Duality and Family Ownership on CSR, suggesting that well governed firms will be more socially responsible. In addition, the findings suggest the institutional quality's positive moderating role on the relationship between ownership structure and CSR, signifying the institutional quality's complementary role for the weak corporate environment in Pakistan. CEO duality negatively affect the level of CSR reporting and performance, these findings are in line with the prior study of Cabeza-García, Sacristán-Navarro [17]. After the introduction section, furthermore the article is organized as: Fig. 1 displays the conceptual framework, The second portion shows theoretical analysis and hypothesis development. The third portion is about the research design, which describes the sample of the study as well as the variables measurement. The fourth section presents the empirical analysis of the study. The last section is about the Discussion, conclusion, future recommendations, and limitations of the study.

## 2 Theoretical analysis and hypothesis development

### 2.1 CEO duality and corporate social responsibility

Excessive power with one individual holding the most important position in the company concerning decision-making is an important issue of corporate governance. The amount of attention paid to CSR disclosure is not dependent on role duality as many companies are paying a significant amount of attention to CSR issues even though they have the person working in both the position of CEO and Chairman. Furthermore, CEO Duality and CSR disclosure are positively related. Conversely, according to agency theory, there should be different people in the role of Chairman and CEO because it will guarantee that the policies and decisions of the management are not abrupt [18]. According to Blackburn [19] if there is role duality it can lead to compromised performance as the CEO will interfere, and the monitoring role of the board may be compromised. According to Petra [20] in light of the agency theory, there will be more decision-making problems as a result of duality and less attention will be paid to the CSR initiatives. Hence, duality will negatively affect the level of CSR reporting and performance [21,22]. Based on the arguments made above and considering the corporate governance and political environment of Pakistan we propose.

H1 There is a significant negative influence of CEO Duality on CSR

### 2.2 Family ownership and corporate social responsibility

A company that is mainly owned by a group of individuals who belong to the same family is referred to as a family-owned firm [23,24]. The boards of such companies are composed of members from the same family who certainly have their own vested interests which are usually different than those of any other organization, Hence they do not engage in CSR activities as much as other firms do [25]. Family-owned companies generally do not pay much attention to improving their relationship with their stakeholders [25].

According to agency theory, family-owned firms should have fewer agency problems [26]. As the family has a major ownership proportion, and prefers strong check and balance over management in order to keep operations smooth [27]. According to Cabeza-García, Sacristán-Navarro [17] CSR disclosure is negatively affected by family ownership. El Ghoul, Guedhami [28] found negative association between family ownership and CSR performance. However, some researchers found that CSR is positively influenced by family ownership. So, in accordance with the weak legislative corporate environment of Pakistan where it is easy to overshadow the views of minority shareholders for personal benefits, as is in the case of family-owned firms, we hypothesized that.

H2 There is a significant negative influence of family ownership on CSR

### 2.3 Foreign ownership and corporate social responsibility

The percentage of stocks owned by foreign shareholders in a company. Foreign investors are believed to be different from local investors in terms of perspective, and the amount of information they have; their choices are based on a completely different understanding as they belong to a different environment [29,30]. According to Khan, Muttakin [31], foreign owners are likely to be more considerate towards social issues as they acquire a better and more informed perspective, this is also in line with legitimacy theory. Increased CSR is deemed to be an instrument that can not only help in reducing information asymmetry but risk as well [32].

In the Asian context, prior studies argued that foreign ownership is positively related to CSR performance [33]. The effect of foreign investors, particularly from North America and Europe, where Corporate Social Responsibility is realized as essential [34]. Better CSR arrangement may result as a significant signaling tool to decrease the issue of information asymmetry between the management and foreign investors [32]. Foreign investors have distinctive investment inclinations as compared to local investors, they evade firms with low disclosure and desire to invest in firms about which they have more clear information [35]. when a firm has foreign ownership the level of its CSR disclosure increases regardless of the fact that the firm operates in an environment where disclosing CSR is voluntary or mandatory [36]. Therefore, based on ground realities and past studies, we hypothesized that.

H3 There is a significant positive impact of foreign ownership on CSR.

### 2.4 Institutional ownership and corporate social responsibility

Institutional investors generally have a hefty investment in the stocks of the company therefore they are not only concerned with the financial but social performance of the company as well. In order to protect their interests, they keep strong control over the management and also take an interest in increasing voluntary CSR disclosures [37]. Moreover, according to Turban and Greening [38] institutional investors are keen to work with companies who indulge in a significant amount of social activities because they foresee the long-term benefits of such engagements. According to previous literature institutional ownership and corporate social responsibility are positively associated [39]. Institutional investors are keen to finance in social ventures to differentiate them from other investors so that their customers are satisfied with them and they can enjoy the continued trust and support from their customers in form of business [39,40]. This is also the idea of legitimacy theory. Hence, according to legitimacy theory and on the basis past of studies we hypothesized that.

H4 There is a significant positive impact of Institutional ownership on CSR

### 2.5 Institutional quality, ownership structure and CSR

The companies which discloses more information regarding their CSR practices are the ones that participate in CSR more as compared to the ones who report relatively less information for CSR [41]. Whereas, Sutantoputra [42] believe that companies disclose their CSR information because they are under some obligation which forces them to report their relevant information in the annual or sustainability report. Hu, Zhu [43] tried to analyze the relationship between ownership pattern and its influence on CSR reporting. Moreover, Elgergeni, Khan [44] stated positive significant effect of ownership structure on CSR.

Institutional quality positively influences the CSR practices in host countries. IQ influences CSR through the relationship between parent and subsidiary firms [45,46]. Institutionally strong and well governed countries are likely to have better CSR to meet the expectations of the meet stakeholders [47]. Although, the complementary notion of resources is principally suggested for companies-level research [48]. Krishnan and Teo [14] expanded its primary argument to the state level study and documented its efficiency. according to them, as complementary assets of the country level, we used the IQ index developed through PCA comprised of governance indicators that will influence the relationship between ownership structure and CSR. According to conversion concept of Lavin, Shapiro [49], we posture that IQ enhances the resources ‘conversion (ownership structure) to production (CSR). The study analyzes the function of IQ for enhancing CSR in a very weak corporate governance environment by following the complementary view of assets. hence, according to the above arguments, we assumed that.

H5a Institutional quality positively moderates the relationship between family ownership. and CSR.

H5b Institutional quality positively moderates the relationship between foreign ownership and CSR.

H5c Institutional quality positively moderates the relationship between institutional ownership and CSR.

With reference to the framework in Fig. 1, this study examines the impact of CEO Duality and ownership structure on CSR; ownership structure is further classified into family ownership, FOW and IOW as these are the prevalent types of ownership in Pakistan, and institutional quality is the moderating variable of the study.

## 3 Methodology

### 3.1 Data and sample

The key objective of the study is to investigate the moderating effect of institutional quality on the association between ownership structure and CSR of the top-performing firms (Based on market capitalization). The data is collected manually from the annual reports of the listed companies on the Pakistan Stock Exchange (PSX), which CSR data is available in the annual reports for the period of (2010–2019). The total listed firms having CSR data were 149, We excluded (27) financial firms which are different in nature than the non-financial firms in terms of regulations. Hence, due to CSR data availability our sample consists of 112 top-performing non-financial firms based on market capitalization for the period of (2010–2019). The firm-year observations are 1079. Missing data firms were omitted, the final sample was comprised of 941 observations. Institutional Quality data is gathered from the portal of World Bank. World governance Indicators are used to measure IQ. Table 1 displays the measurement and acronyms of the variables.

### 3.2 Why Pakistan?

The purpose of the study is to examine if the relationship between ownership structure and CSR is moderated by institutional quality moderates. To the best of knowledge, this issue is not yet focused in the literature of finance. According to the regulatory and institutional differences, Emerging markets like Pakistan characterize an alternate significant setting to investigate this issue for numerous logics. IQ is calculated via an index comprised of six governance indicators. In 2023 report of the transparency international the corruption index of Pakistan is ranked at 133rd position out of 180 as compared to other countries. There is lake of political stability in Pakistan, so none of the prime minister has completed his term from 1947 till today. Even now acting prime minister is ruling the country. Second, the unique and concentrated ownership structure of the firms in Pakistan. In a bid to find out whether firms with different types of ownership structure, which enable them with different level of power in decision making, respond to the social issues in the same way or not. This solemnly depends on the way they disclose this information while preparing their financial statements as they are not obliged to do so in the case of Pakistan. Moreover, this study also takes into account the role of duality as it is quite prevalent in Pakistan [50], but according to the corporate governance code regulations 2017 Chairman and CEO can't be the same person.

### 3.3 Variable measurement

#### 3.3.1 Dependent variables

CSR is measured via two proxies i.e., disclosure and performance. The CSR performance is measured via donation expense in terms of corporate philanthropy. We measure donation using two variables: CSR_D1 and CSR_D2 [51]. CSR_D1 is the total yearly donation expense of the firm divided by total assets and then multiplied by 10,000; this has been multiplied by ten thousand so that the value is not very small however this does not affect the data structure or the results of regression [51]. CSR_D2 is the natural logarithm of total donation expenses. Whereas CSR disclosure is measured via CSR score based on a checklist made from the annual reports with the help of items signified by the SECP and used by past researchers [21,[52], [53], [54], [55]]. A dichotomous approach is used and allotted 1 score if the company has included the relevant item from the checklist otherwise 0 score was given according to Table 1 of the appendix. By following Haniffa and Cooke [21], the CSR index is calculated as follows:

$$CSRD_{j}\;Index=\frac{\sum_{t=1}^{n_{j}}X_{ij}}{n_{j}}$$

where:

**Table 1:** Variable measurement.

Variables	Acronym	Measurement
Dependent Variable		
Corporate Social Responsibility	CSR_Score	Content Analysis on the basis of the CSR Disclosure Index [54]
	CSR_D1	D1:Donation expense/Total Assets*10000 [51]
	CSR_D2	D2: Natural log of donation expense [51]
**Independent Variables**		
CEO Duality	CD	Dummy Variable equals 1 if CEO & Chairman is the same, otherwise 0 [56]
Ownership Structure	FAOW	Dummy Variable equals 1 when the family holds more than 20 % shares, otherwise 0 [57]
	FOW	Binary Variable equals 1 when the foreigners have above than 10 % of shares, otherwise 0 [58]
	IOW	Binary Variable equals 1 when the institutional owner holds more than 10 % of shares, otherwise 0 [57,59]
**Moderator**		
Institutional Quality	IQ_Index	Rule of law, Political stability, regulatory quality, government effectiveness, voice and accountability and control of corruption
**Control Variables**		
Board Independence	BI	Ratio of independent directors to total [31]
Board Size	BSZ	No. of directors on board [54]
Firm Size	FSE	Natural log of total assets
Leverage	LEV	Total debts/Total assets [60,61]
Profitability	ROA	Net Income divided by total assets [51]

CSRD*j* index = CSR Disclosure index for *j*th firm,

n*j* = No of items estimated for *j*th firm, where n ≤ 30,

y = if *i*th items are disclosed is 1 *and* 0 otherwise so that 0 ≤ CSRD*j* ≤ 1.

#### 3.3.2 Independent variables

CEO Duality and Ownership Structure are variables of interest in the study. CEO Duality is measured by using a dummy variable that is equal to 1 if the chairman and the CEO is the same person and otherwise 0 [56]. Moreover, ownership structure is measured with the help of several prevailing types of ownership structures in Pakistan as follows.

a) Family Ownership (FAOW)

Many companies in Pakistan tend to have family-based ownership. Following [57] family ownership is an independent variable measured via a dummy which equals 1 in case the family owns 20 percent or more shares and is the largest shareholder of the firm otherwise 0 [57].

b) Foreign Ownership (FOW)

Foreign ownership is disclosed in the shareholding pattern of the company in its financial statement in Pakistan. So, foreign ownership is calculated by a dummy variable equal to 1 in case 10 percent or more shares are owned by foreign investors otherwise 0 [58].

c) Institutional Ownership (IOW)

The type of ownership where stocks are held by institutions comprised of mutual funds, insurance corporations, pension finances, and investment firms. Institutions usually hold large number of shares of the company to influence the executive. IOW is measured with the help of a dummy, equals to 1 in case the shares’ percentage held by institutions is equal to or more than 10 percent otherwise 0 [57,59].

#### 3.3.3 Moderator (institutional quality) (IQ_Index)

Institutional quality is the moderating variable, comprised of six world governance indicators, i.e, control of corruption, Voice and accountability, rule of law, regulatory quality, government effectiveness and political stability [62,63].

#### 3.3.4 Control variables

Control variables used in this study are firm size, leverage, profitability, board size, and board independence.

### 3.4 Research model

In accordance with Haniffa and Cooke [21] the following model is used

(1) $${CSR}_{it}=\beta_{0}+\beta_{1}{CD}_{it}+\beta_{2}{FAOW}_{it}+\beta_{3}{FOW}_{it}+\beta_{4}{IOW}_{it}+\beta_{5}Controls+\mu_{it}$$

Where:

CSR is the dependent variable, β_0_ is the intercept. Independent variables are CEO Duality (CD), Family ownership (FAOW), Foreign Ownership (FOW), Institutional Ownership (IOW) and Control variables.

To investigate institutional quality's moderating role on the relationship between ownership structure and CSR, we used the following models,

(2) $${CSR}_{it}=\beta_{0}+\beta_{1}{CD}_{it}+\beta_{2}{FAOW}_{it}+\beta_{3}{{IQ}_{it}\times FAOW}_{it}+\beta_{4}{FOW}_{it}+\beta_{5}{IOW}_{it}+\beta_{6}Controls+\mu_{it}$$

(3) $${CSR}_{it}=\beta_{0}+\beta_{1}{CD}_{it}+\beta_{2}{FAOW}_{it}+\beta_{3}{FOW}_{it}+\beta_{4}{{IQ}_{it}\times FOW}_{it}+\beta_{5}{IOW}_{it}+\beta_{6}Controls+\mu_{it}$$

(4) $${CSR}_{it}=\beta_{0}+\beta_{1}{CD}_{it}+\beta_{2}{FAOW}_{it}+\beta_{3}{FOW}_{it}+\beta_{4}{IOW}_{it}+\beta_{5}{{IQ}_{it}\times IOW}_{it}+\beta_{6}Controls+\mu_{it}$$

Where:

CSR is the dependent variable measured by CSR performance and donations, independent variables are family, foreign and institutional ownerships and moderating variable is institutional quality denoted by IQ.

## 4 Empirical analysis

### 4.1 Descriptive statistics

Table 2 displays the descriptive statistics for the period of 2010–2019. Which shows the Institutional quality is 12 % which is very low. The descriptive statistics shows the CSR score 44 % with the standard deviation of 0.225 and the CSR donation mean value is 1.611 with the standard deviation value of 1.441. The descriptive statistics shows the family ownership's average score approximately 78 % which shows that most companies in Pakistan have a significant amount of family shareholding. Family ownership's volatility is 41 % in Pakistan which is very high. The average score of institutional ownership in Pakistan is around 60 % which is also quite high, and it shows that many firms in Pakistan have institutional ownership.

**Table 2:** Ownership structure, CEO duality and CSR (descriptive statistics).

Variable	Obs	Mean	Std. Dev.	Min	Max
CSR_Score	1079	0.442	0.225	0.033	1
CSR_D1	1079	1.611	1.441	0	10.346
CSR_D2	1079	7.129	3.529	0	19.429
CD	1061	0.132	0.339	0	1
FAOW	1070	0.779	0.415	0	1
FOW	1079	0.246	0.431	0	1
IOW	1079	0.601	0.49	0	1
IQ_Index	1079	0.1229103	0.985913	−1.302212	1.371409
BI	1079	0.188	0.175	0	1
BSZ	1079	8.221	2.105	0	21
FSE	1079	16.115	2.982	0	20.457
LEV	1079	0.503	0.245	0	2.103
ROA	1079	0.071	0.088	−0.476	0.67

### 4.2 Ownership structure, CEO duality and CSR disclosure & performance (pooled regression model)

Table 3 presents the results of pooled regression for the negative association between CEO duality and CSR. This means that according to agency theory, there will be more decision-making problems because of duality and less attention is paid to CSR initiatives. Hence, duality negatively affects the level of CSR reporting and performance. Our results are consistent with [21,22]. Foreign ownership was associated with the low level of CRS and family ownership and foreign ownership were positively associated with CSR. However, to confirm that there is no problem of endogeneity and heteroscedasticity, we further applied two-stage least square estimation.

**Table 3:** Ownership structure, CEO duality and CSR disclosure & performance (pooled regression model).

Variables	CSR_Score	CSR_D1	CSR_D2
CD	−0.0612^a^	−0.268	−0.167
	(0.0186)	(0.193)	(0.313)
FAOW	−0.0823^a^	−0.242	−0.351
	(0.0159)	(0.171)	(0.267)
FOW	0.0984^a^	0.263*	0.672^a^
	(0.0148)	(0.157)	(0.249)
IOW	0.0435^a^	0.0392	0.324
	(0.0129)	(0.135)	(0.216)
BI	0.120^a^	−0.252	−0.776
	(0.0362)	(0.389)	(0.607)
BSZ	0.0145^a^	0.119^a^	0.0103
	(0.00353)	(0.0427)	(0.0592)
FSE	0.0185^a^	−0.325^a^	0.113
	(0.00269)	(0.0550)	(0.0452)
LEV	0.00743	−0.475^a^	−0.545^a^
	(0.00767)	(0.0856)	(0.129)
ROA	0.317^a^	6.707^a^	7.095^a^
	(0.0716)	(0.783)	(1.201)
Constant	−0.0317	6.677^a^	7.065^a^
	(0.0587)	(0.902)	(0.984)
Observations	1052	910	1052
R-squared	0.209	0.151	0.071

Standard errors in parentheses.

a ***p < 0.01, **p<0.05, *p<0.1

### 4.3 Two stage lease square (2SLS)

Apart from pooled regression, 2SLS regression has been used to measure the impact of CEO Duality and Ownership Structure on CSR. Moreover, to deal with the problem of endogeneity, 2SLS estimation's linear simultaneous equations is used [64]. Sargan test has been applied to deal with the selection biases of instrumental variables. Every model has been tested to ensure that the instrumental variables that have been used are well specified with the help of Sargan test. According to Wooldridge, Hanson [65] robust score test and robust regression test -based test, we have tested family ownership as endogenous variable and the foreign and institutional ownerships as exogenous. In all cases if the test statistic is significant then the variables test must be treated as endogenous. And the statistics are reported in appendix II.

Table 4 demonstrates CSR score, CSR D1 and CSR D2 as dependent variables, and the independent variables are CD, FAOW, FOW and IOW. Control variables include BI, BSZ, FSE, LEV, and ROA. Family ownership is an endogenous variable in the model which is instrumented by the Lagged value of FAOW and Foreign shares by following the [66,67]. Table 4 depicts the effect of Ownership structure (OS) and CEO Duality on CSR Score and performance, which shows CD and FAOW have a significant and negative association with CSR in Pakistan in light of the agency theory. These results are in line with prior literature of [21,22]. This is also consistent with the respective hypotheses 1 and 2 that According to the result in Table 4 and it is evident that Foreign Ownership is positive and significantly related to CSR which is also consistent with the respective hypotheses 3 and 4 in view of legitimacy theory. Furthermore, to overcome the selection biases of instrumental variables, the over-identification test of the Sargan statistic was applied which confirms that instrumental variables in this model are well specified as the Sargan value is more than 10 %. Our results are consistent with [68]. After controlling for industry fixed effect, the results remain consistent. these results are also consistent with the previous studies such as [32,69].

**Table 4:** Impact of CEO duality and ownership structure on CSR (2SLS) regression.

Variables	CSR_Score	CSR_Score	CSR_D1	CSR_D1	CSR_D2
**FAOW**	−0.082^a^	−0.0670^a^	−0.343^a^	−0.733	−0.655^a^
	(-5.550)	(-4.550)	(-2.272)	(-2.438)	(-2.251)
**CD**	−0.066^a^	−0.0388^a^	−0.458^a^	−0.00625	−0.339^a^
	(-3.830)	(-2.721)	(-2.213)	(-1.774)	(-1.926)
**FOW**	0.094^a^	0.0610^a^	0.572^a^	0.594	0.703^a^
	(6.950)	(4.094)	(3.385)	(2.204)	(2.790)
**IOW**	0.046^a^	0.0253	0.084^a^	0.615^a^	0.342
	(3.640)	(1.934)	(2.897)	(3.004)	(1.685)
**BI**	0.592^a^	0.00871	0.151^a^	−2.513	0.542
	(2.766)	(2.321)	(3.210)	(-1.983)	(2.723)
**BSZ**	0.016^a^	0.0198^a^	0.117	0.130	0.001
	(4.370)	(5.438)	(2.925)	(1.682)	(0.014)
**FSE**	0.019^a^	0.0124	−0.326^a^	0.248^a^	0.111
	(4.370)	(1.564)	(-5.433)	(3.807)	(1.881)
**LEV**	0.009	−0.098^a^	−0.479^a^	−0.312	−0.553^a^
	(1.080)	(-3.160)	(-4.562)	(-1.793)	(-2.926)
**ROA**	0.313^a^	0.0454	6.721^a^	5.601^a^	7.122^a^
	(4.710)	(0.625)	(8.059)	(4.167)	(5.852)
Constant	−0.031	0.143	6.680^a^	3.981^a^	7.059^a^
	(-0.400)	(1.782)	(7.341)	(2.954)	(5.515)
Mean dependent var	0.442	0.622	0.984	0.743	7.185
R-squared	0.201	0.424	0.150	0.222	0.069
Industry FE	NO	Yes	NO	YES	NO
First stage F-test	35.125	32.160	22.170	21.430	9.946
Sargan Test Values	5.535	5.734	6.250	6.340	6.138
Number of Companies	112	112	112	112	112
SD dependent var	0.225	0.312	2.127	2.342	3.480
Number of obs	1052	1052	910	1052	052
Prob > F	0.000	0.000	0.000	0.000	0.000
Prob	0.1959	0.1712	0.1441	0.1231	0.114

Note: Instrumented: FAOW.

Instruments: CD, FOW, IOW, COW, BSZ, FSE, LEV, ROA, FAOW.

a ***p < 0.01, **p<0.05, *p<0.1

### 4.4 Institutional quality, ownership structure, and CSR (2SLS regression)

Table 5 shows the two SLS results for the moderating role of Institutional quality on the relationship between ownership structure and CSR. Table 5 shows the interaction term of IQ and FAOW is positively related with CSR score and CSR D1 and CSR D2. The results also show that interaction term of IQ and FOW and the interaction term of IQ and IOW with CSR is positively related. Hence, the results in Table 5 shows that the IQ positively moderate the relationship between ownership structure and CSR which affirms the hypotheses 5a, 5b and 5c of the study.

**Table 5:** Institutional quality, ownership structure and CSR (2SLS estimation).

VARIABLES	CSR_Score	CSR_D1	CSR_D2	CSR_Score	CSR_D1	CSR_D2	CSR_Score	CSR_D1	CSR_D2
IQP_Index X FAOW	0.190^a^	0.451^a^	0.519^a^						
	(4.029)	(4.282)	(2.825)						
IQP_Index X FOW				0.00961	0.424^a^	0.524^a^			
				(1.228)	(3.760)	(2.863)			
IQP_Index X IOW							0.0110	−0.296^a^	0.220^a^
							(1.357)	(-3.625)	(3.535)
IQP_Index	−0.102^a^	−0.0356	−0.127	0.0462^a^	0.148	0.471^a^	0.0401^a^	0.101	0.433^a^
	(-2.699)	(-0.770)	(-1.678)	(7.284)	(1.869)	(3.748)	(4.648)	(0.996)	(2.828)
FAOW	−0.0886^a^	−0.368	−0.409	−0.0387^a^	−0.273	−0.351	−0.0388^a^	−0.275	−0.456
	(-4.024)	(-1.318)	(-1.005)	(-2.754)	(-1.374)	(-1.240)	(-2.776)	(-1.384)	(-1.389)
FOW	0.0738^a^	0.0128	0.739^a^	0.0716^a^	0.169	0.664^a^	0.0723^a^	0.172	0.711^a^
	(4.493)	(0.0649)	(2.654)	(4.970)	(0.891)	(2.668)	(4.983)	(0.896)	(2.660)
IOW	0.0240	0.379	0.711^a^	0.0278	−0.341	−0.409	0.0270	−0.347	−0.595^a^
	(1.653)	(2.521)	(3.295)	(2.201)	(-2.451)	(-1.993)	(2.152)	(-2.495)	(-2.880)
CD	−0.0349	0.0719	−0.0760^a^	−0.0334	−0.132	0.191	−0.0333	−0.133	0.0929
	(-1.790)	(1.010)	(-2.668)	(-2.219)	(-0.594)	(0.688)	(-2.217)	(-0.600)	(0.322)
Constant	0.184	6.662^a^	4.164^a^	0.162	7.246^a^	7.061^a^	0.161	7.270^a^	4.168^a^
	(2.201)	(4.915)	(3.001)	(2.155)	(5.418)	(5.600)	(2.154)	(5.438)	(3.188)
Observations	941	817	941	1052	910	1052	1052	910	1052
R-squared	0.361	0.254	0.260	0.461	0.239	0.097	0.461	0.239	0.239
Controls	Yes	Yes	Yes	Yes	Yes	Yes	Yes	Yes	Yes
Industry FE	Yes	Yes	Yes	Yes	Yes	Yes	Yes	Yes	Yes

Robust t-statistics in parentheses.

a ***p < 0.01, **p<0.05, *p<0.1

#### 4.4.1 Institutional quality index (IQ)

The IQ index is established through PCA comprised of six WGI, i.e, government efficiency, control of corruption, regulatory quality, political stability, voice and accountability and rule of law. All variables contribute positively to the index., by following [70] two concerns have been addressed before reporting the index. We have used Kaiser–Meyer–Olkin (KMO) test value is 0.511 to ensure the correlation among variables is higher than the correlation among the errors, and Bartlett's test for sphericity is used to ensure that variables are factorable as (P-value<00.001) reported in appendix Table III.

### 4.5 Robustness checks (GMM estimation)

#### 4.5.1 Ownership structure, CEO duality and CSR (GMM) regression

Table 6 displays the results for GMM regression, which are robust and consistent with the main results for (CD), (FAOW), (FOW) and (IOW) with CSR disclosure and Performance. According to the results in Table 6, CD and FAOW are significantly and negatively associated with the lagged value of CSR_D1. Moreover, FOW and IOW are positively and significantly linked to the lagged value of CSR_D1 according to legitimacy theory foreign investors will place considerable importance on the social behavior of the company surety of the business operations, which is in line with the previous literature of [17]. Also, according to Hansen test the model is valid and robust.

**Table 6:** Ownership structure, CEO duality and CSR (GMM) regression.

Variables	CSR_Score	CSR_Score	CSR_D1	CSR_D1	CSR_D2
**LagCSR_D1**	0.456^a^	−0.238^a^	−0.406^a^	−0.238^a^	−1.470^a^
	(8.380)	(-4.579)	(-0.546)	(-4.579)	(-4.016)
**CD**	−0.196^a^	−0.00739	−0.952^a^	−0.00739	−3.611^a^
	(-3.320)	(-1.826)	(-2.712)	(-1.826)	(-3.446)
**FAOW**	−0.299^a^	−23.34^a^	−1.004^a^	−23.34^a^	−0.357^a^
	(-2.570)	(-4.548)	(-1.884)	(-4.548)	(-5.578)
**FOW**	0.173^a^	0.127	0.558^a^	−23.34^a^	0.431
	(3.380)	(1.526)	(2.335)	(-4.548)	(2.082)
**IOW**	0.277^a^	0.320	0.850^a^	0.320	0.743
	(5.320)	(0.394)	(2.146)	(0.394)	(5.992)
**BI**	0.366	−1.758	−2.513	0.222	−0.020
	(2.270)	(-0.587)	(-1.983)	(1.476)	(-0.007)
**BSZ**	0.058^a^	−0.244	−0.466^a^	−0.00228	−0.469
	(3.180)	(-0.594)	(-2.824)	(-0.101)	(-1.439)
**FSE**	−0.004	−2.753^a^	0.419^a^	0.00296	0.176
	(-0.420)	(-5.870)	(2.074)	(0.235)	(1.135)
**LEV**	−0.098^a^	−0.885	0.643	0.00749	0.331
	(-3.160)	(-1.731)	(2.165)	(0.248)	(0.604)
**ROA**	0.528	21.32^a^	4.812	0.103	10.714
	(1.960)	(3.725)	(1.702)	(0.291)	(2.243)
Constant	−0.288	65.24^a^	−3.225	−0.0742	8.309
	(-1.020)	(6.555)	(-0.762)	(-0.275)	(1.654)
Industry FE	NO	YES	NO	YES	NO
Mean dependent var	0.450	0.421	1.014	1.123	7.270
Number of obs	941	941	817	817	941
Number of Companies	112	112	112	112	112
AR(1)	−8.67	−7.51	−4.79	−3.89	−5.57
AR(2)	−1.26	−1.11	−2.35	−2.13	−2.68
Hansen Test	8.509	8.132	9.18	8.78	8.79
Number of Instrument	32	32	32	32	32
SD dependent var	0.224	0.219	2.104	2.109	3.483
Chi-square	13054.9	13021.1	650.15	620.15	10590.286
Prob.	0.000	0.000	0.000	0.000	0.000
Prob.	0.207	0.210	0.319	0.219	0.207
Prob.	0.448	0.324	0.178	0.165	0.183

a ***p < 0.01, **p<0.5, *p<0.1

After controlling industry, the findings remain constant with the main results and parallel to the previous literature of [17].

#### 4.5.2 Institutional quality, ownership structure and CSR (GMM) regression

Table 7 shows the robust results for the moderating role of institutional quality on the relationship between ownership structure and CSR. Table 7 shows the positive relationship of the interaction term of IQ and FAOW with CSR. The results also found that the interaction term of IQ and IOW and the interaction term of IQ and FOW positively related with CSR. These results affirm the main results and show that the IQ positively moderates the relationship between ownership structure and CSR.

**Table 7:** Robust checks: Institutional Quality, Ownership Structure and CSR (GMM).

VARIABLES	CSR_Score	CSR_D1	CSR_D2	CSR_Score	CSR_D1	CSR_D2	CSR_Score	CSR_D1	CSR_D2
**Lag CSR_Scor**	0.971			−0.915			−0.804		
	(1.811)			(-1.752)			(-1.944)		
**Lag CSR_D1**		−0.233^a^			−0.227^a^			−0.246^a^	
		(-4.359)			(-4.242)			(-4.584)	
**Lag CSR_D2**			−0.223^a^			−0.201^a^			−0.259^a^
			(-5.304)			(-4.334)			(-5.751)
**IQ_Index X FAOW**	0.455	2.884^a^	−1.177						
	(2.203)	(5.440)	(-1.258)						
**IQ_Index X FOW**				0.00330	0.915	2.840			
				(0.100)	(1.752)	(1.962)			
**IQ_Index X IOW**							0.913	−0.189	3.386
							(1.778)	(-0.210)	(2.409)
**IQ_Index**	0.0446	0.575	0.919	0.00330	0.915	0.790	0.00488	0.913	2.075
	(0.738)	(0.728)	(1.227)	(0.100)	(1.752)	(1.853)	(0.145)	(1.778)	(2.363)
**FAOW**	0.399	−22.82^a^	5.448	0.316	−24.26^a^	6.937^a^	0.326*	−23.33^a^	7.808^a^
	(2.013)	(-4.285)	(2.465)	(1.874)	(-4.569)	(3.919)	(1.896)	(-4.562)	(2.958)
**FOW**	−0.0453	−0.163	−0.112	−0.0424	−0.737	−3.069	−0.0442	−0.275	−1.418
	(-0.604)	(-0.149)	(-0.0783)	(-0.572)	(-0.643)	(-1.553)	(-0.594)	(-0.269)	(-0.879)
**IOW**	−0.0107	0.475	−1.386	−0.00777	0.429	−1.422	0.000736	0.548	−0.236
	(-0.188)	(0.559)	(-1.176)	(-0.138)	(0.516)	(-1.151)	(0.0108)	(0.654)	(-0.173)
**CD**	−0.406^a^	0.602	−1.740	−0.404^a^	0.542	−1.408	−0.404^a^	0.536	−1.974
	(-4.781)	(0.553)	(-1.231)	(-4.778)	(0.501)	(-0.943)	(-4.821)	(0.503)	(-1.292)
**Constant**	−0.119	66.47^a^	−15.98	−0.0590	68.66^a^	−17.03	−0.0898	68.44^a^	−14.34
	(-0.403)	(6.181)	(-2.217)	(-0.209)	(6.362)	(-2.176)	(-0.288)	(6.490)	(-1.793)
**Observations**	941	772	941	941	772	941	941	772	941
**Number of Firms**	111	110	111	111	110	111	111	110	111
**Controls**	Yes	Yes	Yes	Yes	Yes	Yes	Yes	Yes	Yes
**Industry FE**	Yes	Yes	Yes	Yes	Yes	Yes	Yes	Yes	Yes

z-statistics in parentheses.

a ***p < 0.01, **p<0.05, *p<0.1

## 5 Discussion

Corporate Social Responsibility is getting more importance in Pakistan than it used to get in other parts of the world but still it is not a mandatory reporting requirement. Many firms are still not taking part in CSR. They are not conscious of the advantages of CSR and therefore believe that it is an additional burden on their finances. Moreover, a considerable number of companies in Pakistan perceive CSR as only limited to donations and hence to fulfill their CSR objective they participate in a different form of donations. The key purpose of this Study is to investigate if IQ complements the relationship among Ownership Structure and CSR performance and disclosure in the light of legitimacy and agency theory. IQ positively influences CSR practices in host countries. First, IQ influences CSR through the relationship between parent and subsidiary firms and finds that CSR is positively influenced by IQ's pressure [45]. Our results are consistent with the respective hypotheses 1 and 2 that CD and FAOW have a significant and negative association with CSR in Pakistan. According to the agency theory there will be more decision-making problems because of duality and less attention will be paid to the CSR initiatives. Duality and family ownership negatively affect the level of CSR reporting and performance, foreign ownership can increase stakeholder diversity, as the company may need to respond to the interests and expectations of a broader range of stakeholders. To check the robustness of our 2SLS method's results, two-step GMM is used to derive regression results with corrections and to check the validity of an instrument Hansen test is used. By following Roodman [71] latest techniques are used to solve the problem of over-identification which are because of the proliferation of instruments (the bias of too many instruments). Excluding data that has extreme values in the first or last period, the smaller data set can be used to conduct a robustness check. The Hansen test is used to tests the validity of the specified model. To analyze correlation among the error term and instruments. The AR (2) second-order autocorrelation test is also reported. Given the use of first-difference transformations, some degree of first-order serial correlation was expected, but the results were not invalidated. According to Table 6, CD and FAOW are negatively and significantly linked to the lagged value of CSR_D2. Agency theory suggests that conflicts of interest can arise between administrators and shareholders, and family ownership can complicate this relationship by introducing additional conflicts between family members and other shareholders. These results remained consistent with the main results. Moreover, FOW is significantly and positively associated with the lagged value of CSR_D2 according to legitimacy theory foreign investors will place considerable importance on the social behavior of the company to ensure continuity of business operations.

## 6 Conclusion

This study is an attempt to analyze the IQ's role for enlightening CSR in a weak corporate governance environment by following the complementary phenomenon of assets. The sample comprises of 112 top-performing listed firms (based on market capitalization) at Pakistan Stock Exchange from 2010 to 2019. Institutional quality comprised of world governance indicators which is developed via principal component analysis, an instrumental variable approach and content analysis are used for CSR Disclosure Index to demonstrate the relationship between ownership structure and CSR. The resources complementary phenomenon is adopted to examine the institutional quality's role. Our results show significantly positive impact of Institutional and Foreign Ownerships on CSR while negative significant influence of CEO Duality and Family Ownership on CSR, suggesting that well governed firms will be more socially responsible. In addition, the findings suggest the institutional quality's positive moderating role on the relationship between ownership structure and CSR, signifying the institutional quality's complementary role for the weak corporate environment in Pakistan. CSR decisions are generally influenced by shareholders and are taken at the board level. Therefore, it is immensely important to find out how different type of ownership impacts the CSR decision. So, this study tries to find out the impact of CEO duality and different types of ownership on CSR decisions in terms of both disclosure and performance. For this purpose, a comprehensive methodology was used. CSR score index was formed in accordance with that of the previous researchers for the CSR disclosure. For CSR performance the donation expense was used in different proxies in accordance with that of the previous researchers to find the impact on CSR performance this was used because in Pakistan there is a strong culture of corporate donations.

The results after accounting for endogeneity and heteroscedasticity were in accordance with the proposed hypotheses of the study. The empirical results suggest that CEO Duality and FAOW have a negative effect on CSR decisions. The empirical results also showed that FOW and IOWs are positively associated with CSR performance as well as CSR disclosure. institutional owners prefer CSR because they see it to enhance the long-term sustainability and profitability of the companies they invest in, while also mitigating potential risks and complying with regulations, and foreign investors prefer CSR activities to mitigate risks that foreign investors face while investing in foreign markets. which states that foreign investors should give considerable importance to the social behavior of the company to make sure the continuity of operations in Pakistani firms. All the empirical findings of this study are robust.

### 6.1 Implications and limitations

The extensive period will permit the study to examine incidents such as the financial crises before 2010 and covid19 after 2019 and first code of corporate governance in Pakistan. Moreover, as CSR has not been well studied in Pakistan and this study only focused on non-financial companies, the future study in comparison with other Asian countries which have similar structures and CSR environments could be of more interest. This study only included CEO duality and 3 types of ownership so future studies can include ownership's types such as state ownership, and managerial ownership. And the future study can also include audit committee characteristics to check the relationship with CSR. Executives, organizations, and stakeholders must be strict in supervising corporate governance arrangements, to ensure laws in corporate environments.

## Funding

This work was supported by Chinese National Funding of Social Sciences ( Grant Number: 20BGL076)

## Data availability statement

The data will be made available on request.

## CRediT authorship contribution statement

**Shuaib Ali:** Writing – review & editing, Writing – original draft, Software, Data curation. **Rong Wu Zhang:** Supervision, Resources, Funding acquisition. **Muhammad Talha:** Writing – review & editing, Investigation, Data curation. **Zahid Ali:** Writing – review & editing, Software, Methodology.

## Declaration of competing interest

The authors declare that they have no known competing financial interests or personal relationships that could have appeared to influence the work reported in this paper.

## Figures and Tables

**Table appendix I:** Checklist of factors included in study

Contribution to Health Sector	
1	Policies for health and safety
2	Assistance program for HIV/AIDS
3	Support to hospitals in terms of medical equipment
4	Support for cancer and other hospitals
5	Establishment of health care center for underprivileged people
**Facilities for Employees**	
1	Medical facility for employees
2	Employee training programs
3	Employee benefits
4	Career development programs
5	Benefits for employees
6	Facilities for employee's family
7	Measures for safety of employees
**Environmental Contribution**	
1	Measures for environment protection
2	Support for tree plantation drives
3	Support for public/private actions designed to protect the environment
4	Promoting environmental awareness through different promotional tools
**Contribution to Education Sector**	
1	Supporting the establishment of research centers at universities
2	Scholarship to research students at universities
3	Internship/Apprenticeship opportunities for students
4	Fundraising/supporting different education programs
5	Fund for special education
**Activities for Community**	
1	Work for physically disabled or especially abled persons
2	Child Labor Policy in order to reduce child labor
3	Donation to relief fund of different calamities (river erosions/floods/earthquake)
4	Work and support for rehabilitation of people affected by natural disasters
5	Support to different NGO's working for uplifting poor people
**Product and Services Statements**	
1	Awards received for CSR activities
2	Information for conducting research on company's products
3	Product Quality Policy
4	Explanation of major project/services/segments/products

**Table appendix II:** test for endogeneity

Variables	Robust Score chi2	P	Robust regression	P
FAOW	5.80883	0.0159	4.68703	0.0306
FOW	1.81122	0.1784	1.83704	0.1756
IOW	2.4428	0.1181	2.44973	0.1179

**Table appendix III:** Institutional quality Index

Variables	Weights
Control of corruption	0.5989
Government effectiveness	0.2086
Political stability	0.5596
Regulatory quality	0.1244
Rule of law	0.4957
Voice and Accountability	0.1530
Kaiser-Meyer-Olkin Statistic	0.511
Bartlett's Chi-square	6598.078
Bartlett's test p-value	0.000
